# Research on the Internal Flow Field of Left Atrial Appendage and Stroke Risk Assessment with Different Blood Models

**DOI:** 10.3390/bioengineering10080944

**Published:** 2023-08-08

**Authors:** Jun Yang, Zitao Bai, Chentao Song, Huirong Ding, Mu Chen, Jian Sun, Xiaohua Liu

**Affiliations:** 1 School of Energy and Power Engineering, University of Shanghai for Science and Technology, Shanghai 200093, China; yangjun@usst.edu.cn (J.Y.); 212210052@st.usst.edu.cn (Z.B.); 203630221@st.usst.edu.cn (C.S.); 2 Xinhua Hospital, School of Medicine, Shanghai Jiao Tong University, Shanghai 200240, China; dinghuirong@xinhuamed.com.cn (H.D.); chenmu@xinhuamed.com.cn (M.C.); 3 School of Aeronautics and Astronautics, Shanghai Jiao Tong University, Shanghai 200240, China

**Keywords:** atrial fibrillation, computational fluid dynamics, left atrium, left atrial appendage, thrombus

## Abstract

Extant clinical research has underscored that patients suffering from atrial fibrillation (AF) bear an elevated risk for stroke, predominantly driven by the formation of thrombus in the left atrial appendage (LAA). As such, accurately identifying those at an increased risk of thrombosis becomes paramount to facilitate timely and effective treatment. This study was designed to shed light on the mechanisms underlying thrombus formation in the LAA by employing three-dimensional (3D) left atrium (LA) models of AF patients, which were constructed based on Computed Tomography (CT) imaging. The distinct benefits of Computational Fluid Dynamics (CFD) were leveraged to simulate the blood flow field within the LA, using three distinct blood flow models, both under AF and sinus rhythm (SR) conditions. The potential risk of thrombus formation was evaluated by analyzing the Relative Residence Time (*RRT*) and Endothelial Cell Activation Potential (*ECAP*) values. The results gleaned from this study affirm that all three blood flow models align with extant clinical guidelines, thereby enabling an effective prediction of thrombosis risk. However, noteworthy differences emerged when comparing the intricacies of the flow field and thrombosis risk across the three models. The single-phase non-Newtonian blood flow model resulted in comparatively lower residence times for blood within the LA and lower values for the Oscillatory Shear Index (*OSI*), *RRT*, and *ECAP* within the LAA. These findings suggest a reduced thrombosis risk. Conversely, the two-phase non-Newtonian blood flow model exhibited a higher residence time for blood and elevated *RRT* value within the LAA, suggesting an increased risk for thrombosis.

## 1. Introduction

Atrial fibrillation (AF) represents the most prevalent cardiac arrhythmia diagnosed in clinical practice [1]. Among the multitude of potential implications associated with AF, an increased predisposition to stroke remains prominent. Statistical data have revealed that a staggering 70 to 90% of AF-related strokes can be traced back to thrombus formation in the left atrial appendage (LAA), a specific cavity within the left atrium (LA). This phenomenon is attributed to the irregular motion of the LA due to AF, leading to decelerated blood flow and subsequent stasis, thereby escalating the risk of thrombus formation within the LAA [2].

In recent years, the LA/LAA hemodynamics have been subjected to extensive analysis by numerous scholars, employing Computational Fluid Dynamics (CFD) as a methodological cornerstone. Qureshi et al. [3] introduced an innovative modeling methodology for quantifying the probability of AF-associated thrombogenesis within the LA, underscoring an elevated thrombus formation risk in the LAA. Concurrently, Zhang and Gay [4] conducted an exploration of AF’s impact on the internal flow within the LA, discovering that the LAA failed to function under sinus rhythm (SR) conditions, thereby precipitating blood stagnation and subsequent thrombosis during AF. Moreover, Olivares et al. [5] utilized the CFD method to evaluate blood flow patterns following LAA occlusion and assessed thrombosis risk by referencing the Endothelial Cell Activation Potential (*ECAP*) value. Subsequently, the same research team [6] employed the Relative Residence Time (*RRT*) value as a measure for characterizing thrombosis risk and concluded that areas proximal to the LAA ostium, as well as those with lobes, exhibited an increased coagulation propensity owing to the presence of low velocities and vortices.

Despite numerous studies focusing on blood flow dynamics and thrombosis risk prediction within the LAA, the blood flow models utilized differ significantly. At present, these primarily include the single-phase (SP) Newtonian, SP non-Newtonian, two-phase (TP) non-Newtonian, and three-phase blood flow models.

For instance, Bosi et al. [7] treated blood as an SP Newtonian fluid to delve into the influence of LAA morphology on hemodynamics under both SR and AF conditions. Andrzej et al. [8] presented a three-dimensional (3D) kinetic model of thrombus formation within an endovascular prosthesis, exploring thrombosis risk by treating blood as an SP non-Newtonian fluid. Furthermore, Qiao et al. [9] developed an aortic dissection model considering blood as a two-phase flow, subsequently exploring the effects of in situ fenestration–thoracic endovascular aortic repair (ISF-TEVAR) operation through the examination of hemodynamic parameters. Zhang et al. [10] conceptualized blood as a TP fluid composed of a primary phase of Newtonian fluid plasma and a secondary phase of pseudo-fluid red blood cells. Additional studies by Jhonston et al. [11] and Hassanein et al. [12] used both Newtonian and non-Newtonian blood flow models to study the wall shear stress (*WSS*) distribution for transient blood flow in arteries, and they used a three-phase CFD model including plasma, red blood cells (*RBC*), and leukocytes to simulate local hemodynamics and track *WSS*, phase distributions, and flow patterns for each phase of blood, respectively. Arzani et al. [13] proposed a novel hybrid Newtonian and non-Newtonian rheology model which shows a significant reduction in shear-thinning effects and provides hemodynamic results that are qualitatively identical and quantitatively close to the Newtonian model, suggesting that non-Newtonian models should be revisited in large artery flows. What is more, Gonzalo et al. [14] modeled the effects of hematocrit and rouleaux formation kinetics by varying the parameterization of the Carreau–Yasuda relation and modulating non-Newtonian viscosity changes based on residence time (*RT*), suggesting that hematocrit-dependent non-Newtonian blood rheology should be considered when calculating patient-specific blood stasis indices via CFD. Lastly, Liu et al. [15] investigated the cerebral hemodynamic metrics discrepancies quantified in CFD models built with Newtonian and non-Newtonian fluid assumptions and concluded that the Newtonian fluid model could be applicable for the pressure ratio calculation. However, they recommended careful consideration while employing the Newtonian assumption for simulating *WSS*, particularly in severe intracranial atherosclerotic stenosis cases.

At this juncture, the scientific community lacks a consensus regarding the optimal blood flow model, with each model demonstrating a specific degree of efficacy across various scales, ranging from large arteries to microcirculation. This necessitates the careful selection of the most fitting model for distinct case scenarios. Consequently, the comparative analysis of different rheological models is critical, as these models could lead to significant variations in hemodynamic parameters, including *WSS* and the low-density lipoprotein (LDL) filtration rate in areas with irregular flow patterns [16]. Despite some research comparing different blood flow models in the aorta, coronary artery, and other components of the cardiovascular system [13,15,16], comparative research focusing specifically on the LA and LAA remains scant [14].

In this study, we reconstructed 3D LA models of three AF patients by using computed tomography (CT) image data. The thrombosis risk and the blood flow distribution in the patients’ LAs, simulated using different blood flow models, were compared and analyzed with the aim of discerning the influence of diverse blood flow models on the simulation outcomes.

## 2. Materials and Methods

### 2.1. Geometric Models

**Figure 1:**
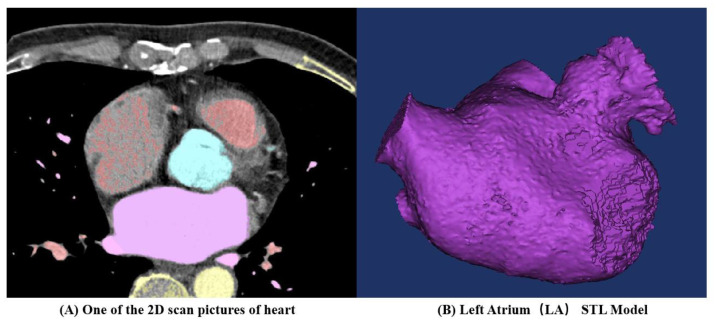
Three-dimensional (3D) left atrium (LA) model reconstruction process for one patient.

**Figure 2:**
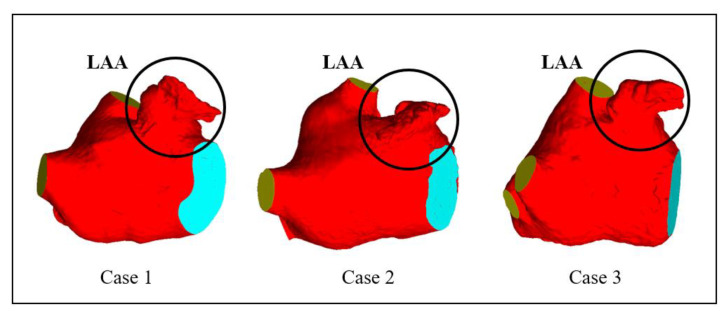
LA 3D models with left atrium appendage (LAA) of 3 patients.

CT images obtained from three volunteers diagnosed with AF, in compliance with the informed consent of each patient, were used in this study. These scans were conducted at the Xinhua Hospital, School of Medicine, Shanghai Jiao Tong University. Based on these images, the 3D representations of the patients’ left atria were constructed. An example of one such CT image is illustrated in Figure 1A. The LAAs of all three patients conform to the ‘chicken-wing’ morphological type, with a slice spacing of 0.45 mm, and slice counts of 396, 376, and 370, respectively. 3D reconstruction software 3D Slicer 5.2.2 was used to segment regions such as the left ventricle, right ventricle, left atrium, right atrium, and aorta. The 3D reconstruction software programs that are commonly used for this are 3D Slicer and Mimics. Utilizing these two-dimensional CT images, three 3D heart models were generated, with one of the resultant STL models showcased in Figure 1B. Figure 2 shows the LA 3D models with LAA of 3 patients. The primary geometric parameters associated with the patients’ LAs are displayed in Table 1. The mitral valve (MV) orifice area corresponds to the area of the flat section derived from slicing the MV orifice on the LA 3D model, using a plane cutting tool. Among all the cases, Case 2 demonstrated atrial enlargement and was the sole case to have experienced a stroke. Interestingly, Case 3 presented with five pulmonary vein (PV) openings, one more than the norm. Previous studies have indicated that over 10% of AF patients exhibit additional PVs [14], displaying a trend of heightened AF frequency [17].

### 2.2. Thrombosis Prediction Model

Existing studies have suggested that thrombus formation is intimately tied to the *RT* of blood components (particles) in close proximity to endothelial cells [18]. Consequently, in this paper, the risk of thrombus formation is represented by the *RRT* value. An escalated risk of thrombosis can be inferred from a higher *RRT* value [19].

*RRT* is intrinsically connected to the Oscillatory Shear Index (*OSI*) and Time Average Wall Shear Stress (*TAWSS*) [20]. *TAWSS* embodies the biomechanical impact of *WSS* on the LA wall. A lower *WSS* value signifies reduced flow velocity, which is related to blood stasis and an elevated risk of coagulation [21]. *OSI*, a dimensionless parameter that ranges from 0 to 0.5, provides insight into the deviation of *WSS* from the dominant blood flow direction during a cardiac cycle. As the value approaches 0.5, the alterations in blood flow patterns become more intricate, thus facilitating thrombus formation. *RRT* integrates *WSS* and *OSI* and can depict the *RT* of blood particles near the wall—the greater the *RRT* value, the higher the probability of thrombus formation in the nearby region.

*TAWSS*, *OSI*, and *RRT* are calculated as follows:

(1) $$TAWSS=\frac{1}{T}\int_{0}^{T}\left|\vec{WSS}\right|dt$$

(2) OSI=0.5×(1−1T|∫0TWSS→dt|TAWSS)

(3) $$RRT=\frac{1}{(1-2\times OSI)\times TAWSS}$$

where *t* is the time, and *T* is the cardiac period.

*ECAP* is another in silico hemodynamic index that combines *TAWSS* and *OSI*, and it is computed as follows [22]:

(4) $$ECAP=\frac{OSI}{\bar{TAWSS}}$$

The *ECAP* hemodynamic index was initially developed to identify regions susceptible to aneurysm thrombus formation [23]. It can detect areas characterized by low blood flow velocities and complex flow patterns. The *ECAP* values in regions with low blood flow velocities (i.e., low *TAWSS* values) and intricate fluid patterns (i.e., high *OSI* values) tend to be higher, indicating an increased risk of thrombus formation.

### 2.3. Solving Process

**Figure 3:**
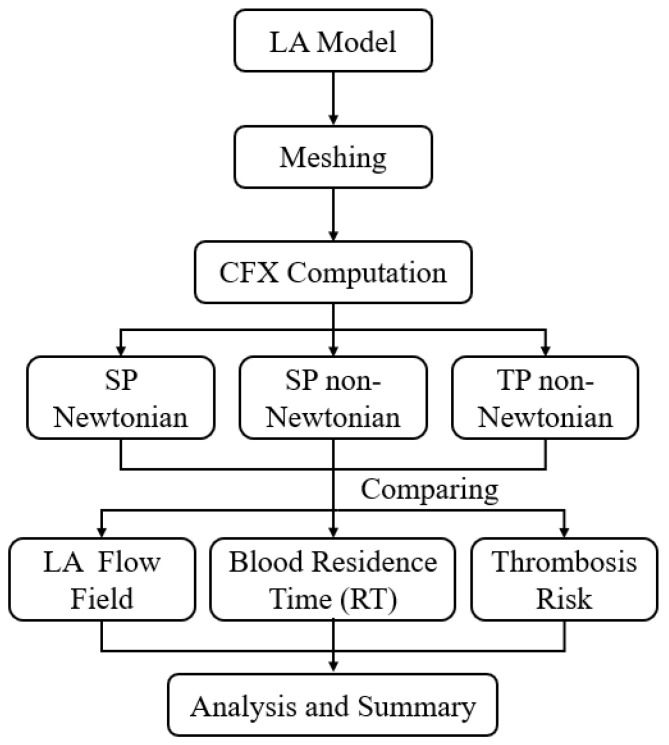
Schematics of the calculation methodology.

In the simulation, blood is alternately considered to be an SP Newtonian fluid, SP non-Newtonian fluid, and TP non-Newtonian fluid. The calculation methodology employed is illustrated in Figure 3. The details of each model are as follows.

#### 2.3.1. Single-Phase Newtonian Blood Flow Model

In both SP Newtonian and non-Newtonian blood flow models, the incompressible Navier–Stokes equation and the continuity equation serve as the basis for modeling, employed to accurately describe the flow dynamics of the blood.

(5) $$\frac{\partial \vec{v}}{dt}+\vec{v}\cdot \nabla \vec{v}=-\frac{1}{\rho }\nabla p+\frac{\mu }{\rho }\nabla^{2}\vec{v}$$

(6) $$\nabla \cdot \vec{v}=0$$

where $\vec{v}$ is the blood flow velocity, *p* is the pressure, and ρ is the blood density.

The blood is set as an adiabatic, incompressible viscous Newtonian fluid. The blood density is 1060 kg/m^3^, and the viscosity is 0.004 Pa·s [24].

#### 2.3.2. Single-Phase Non-Newtonian Blood Flow Model

The blood density is set at 1060 kg/m^3^. Viscosity is a paramount characteristic of any fluid. Research indicates that the non-Newtonian characteristics of blood cannot be disregarded [25]. In the case of the SP non-Newtonian assumption employed in this study, the Quemada model [26] is invoked to represent the non-Newtonian attributes of blood. The Htc value of red blood cell backlog is set at 40%, and the expression is as follows:

(7) $$\eta =\eta_{p}{\left(1-\frac{K\cdot Htc}{2}\right)}^{-2}$$

where η is the blood viscosity, $\eta_{p}$ is the plasma viscosity ($\eta_{p}$= 0.001 Pa·s) [27], *K* is the internal viscosity of red blood cells (calculated from Equation (8)), and Htc is the number of red blood cell backlog (Htc=40%).

(8) $$K=\frac{k_{0}+k_{\infty }{\left(\gamma /\gamma_{c}\right)}^{1/2}}{1+{\left(\gamma /\gamma_{c}\right)}^{1/2}}$$

where $k_{0}$ and $k_{\infty }$ are the parameters to characterize blood behavior ($k_{0}$ = 4.08, $k_{\infty }$ = 1.75) [28], γ is the shear rate, and $\gamma_{c}$ is the critical shear rate.

#### 2.3.3. Two-Phase Non-Newtonian Blood Model

Mass Conservation Equation:

(9) $$\frac{\partial }{\partial t}\left(\varepsilon_{k}\rho_{k}\right)+\nabla \cdot \left(\varepsilon_{k}\rho_{k}\vec{u_{k}}\right)=0$$

where *k* = *l*, *l* is the subscript of plasma, *m* is the subscript of red blood cells, ρ is the density, $\vec{u}$ is the velocity vector, ε is the volume fraction. The sum of the volume fractions of plasma and red blood cells is always equal to 1:

(10) $$\varepsilon_{l}+\varepsilon_{m}=1$$

Momentum Conservation Equation:

(11) $$\frac{\partial }{\partial t}\left(\varepsilon_{k}\rho_{k}\vec{u_{k}}\right)+\nabla \left(\varepsilon_{k}\rho_{k}\vec{u_{k}}\vec{u_{k}}\right)=-\varepsilon_{k}\nabla p+\nabla \cdot {\bar{\bar{\tau }}}_{k}+\varepsilon_{k}\rho_{k}\vec{g}+\sum_{l\neq m}^{n}\beta_{lm}\left(\vec{u_{l}}-\vec{u_{m}}\right)+\vec{F_{k}}$$

where *p* is the pressure; ${\bar{\bar{\tau }}}_{k}$ is the stress tensor; $\vec{g}$ is the gravity; $\beta_{lm}$ is the momentum exchange coefficient between phases; $\vec{F_{k}}$ is the source term including buoyancy force, virtual mass force, and other forces.

Blood is regarded as a TP fluid comprising plasma (the liquid phase) and red blood cells (the solid phase), with plasma and red blood cells constituting 60% and 40% of the total volume, respectively. The plasma density is 1000 kg/m^3^, with a viscosity of 0.001 Pa·s, whereas the red blood cell density stands at 1150 kg/m^3^. Red blood cells are depicted as rigid spherical particles with an 8-micron diameter.

For the TP non-Newtonian assumption in this study, the Carreau–Yasuda model is utilized to emulate the non-Newtonian properties of blood. The dimensionless mixed viscosity can be expressed as follows:

(12) $$\eta =\frac{\sum_{k=1}^{n}\varepsilon_{k}\mu_{k}}{\mu_{plasma}}=m{\left[1+{\left(\lambda \dot{\gamma }\right)}^{2}\right]}^{(n-1)/2}$$

where $\varepsilon_{k}$ and $\mu_{k}$ are, respectively, the volume fraction and viscosity of each phase; $\dot{\gamma }$ is the shear rate; and λ is the time constant (λ = 0.110 s) [27]. Plasma under normal physiological conditions is Newtonian fluid; the plasma dynamic viscosity, $\mu_{plasma}$, is 0.001 kg/(m·s); the *RBC* dynamic viscosity is related to its volume fraction, $\varepsilon_{k}$; and the shear rate, *m*, and *n* are parameters related to the volume fraction of *RBC*.

(13) $$n=0.8092\varepsilon_{RBC}^{3}-0.8246\varepsilon_{RBC}^{2}-0.3503\varepsilon_{RBC}^{}+1$$

(14) $$m=122.28\varepsilon_{RBC}^{3}-51.213\varepsilon_{RBC}^{2}+16.305\varepsilon_{RBC}^{}+1$$

#### 2.3.4. Boundary Condition

In this study, Ansys CFX was utilized for the simulations. The blood flow within the LA was simulated under isothermal conditions. The *RT* serves as a standard measure for the risk assessment of thrombosis, with the *RT* value in this study approximating six cardiac cycles.

To achieve a more stable result, once the calculation result converges, ten cardiac cycles are computed, utilizing the outcomes from the final few cycles to calculate the *RT* value. This approach better illustrates the disparities in *RT* across different sections of the LA. Each cardiac cycle is 0.8 s in duration (comprising 37.5% of atrial diastolic period and 62.5% of atrial systolic period), culminating in a total computation duration of 8 s, with each time step measuring 0.005 s [19]. The convergence criterion is identified as the root mean square residual (RMS), and the residual target is set at 1 × 10^−5^.

**Figure 4:**
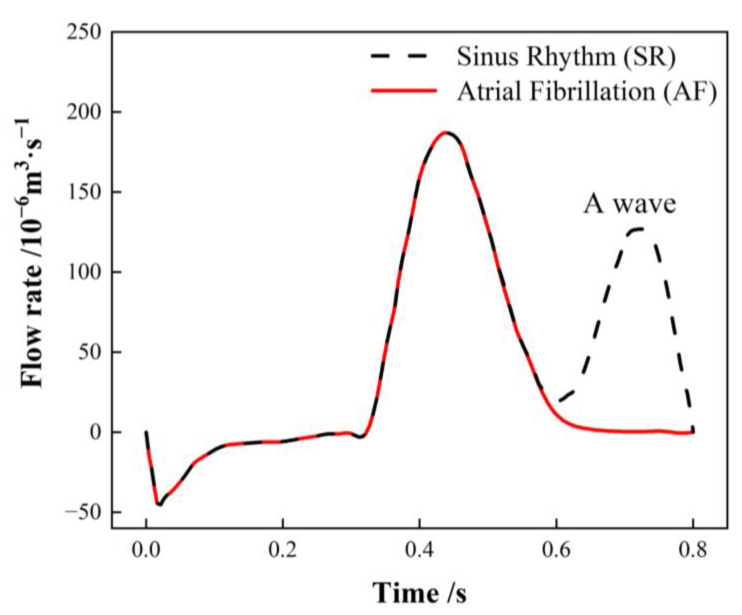
Mitral valve (MV) flow rate in a single cardiac cycle.

The PV inlets across all cases are set to an open boundary condition with a constant pressure of 0 Pa. The flow rate traversing the MV in SR is contingent on the international regulation ISO5840-1:2015 [29], with the flow rate curve for one cardiac cycle illustrated with a black dashed line in Figure 4. The flow rate associated with atrial fibrillation (AF) is calculated by excluding the second atrial emptying wave (A wave) from the healthy mitral blood flow (depicted by the red line) [30]. 

During the simulation, the MV outlet velocity is computed by dividing the MV blood flow by the MV outlet area. A piecewise polynomial function is employed to fit the blood flow velocity waveform, subsequently deriving a blood flow velocity equation for the MV outlet over the course of one cardiac cycle. Due to variations in MV area, the boundary conditions for blood flow velocity differ accordingly. Further details can be referenced in prior research [19].

The occurrence of AF significantly diminishes the contractility of the LAA and LA, rendering the LA walls rigid and inhibiting proper contraction [27]. Numerous researchers have utilized the rigid wall assumption for LAA in simulations under an AF state [7,31,32]. Some scholars have indicated that treating the LA wall as a rigid no-slip wall can simulate the worst case of AF, where there is virtually no contraction of the LA [6]. Consequently, the LA and LAA walls in this study were designated to be rigid no-slip walls.

The additional variable *RT* is modeled as a tracer passively transported with the flow, and its trajectory is determined by the transport equation to represent the *RT* of blood in the LA region. The initial value of *RT* is established at zero, with the transport equation being as follows [33]:

(15) $$\frac{\partial RT}{\partial t}+v\cdot \nabla RT=D_{RT}\nabla^{2}RT+1$$

where *t* is the time, *v* is the blood flow speed, $D_{RT}$ is the self-diffusion rate of blood ($D_{RT}$ = 1.14 × 10^−11^ m^2^/s) [33], and the source term “1” considers a unit increase in *RT* for each unit increase in time.

### 2.4. Blood Flow Model Verification

To validate the precision of the three proposed blood flow models, the classic experiment on blood flow within a tubular sudden expansion channel, as conducted by Karino [34], was selected for numerical verification.

**Figure 5:**
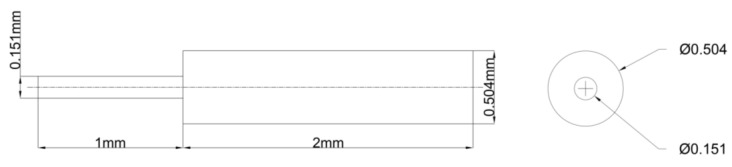
The geometry of the tubular sudden expansion channel.

**Figure 6:**
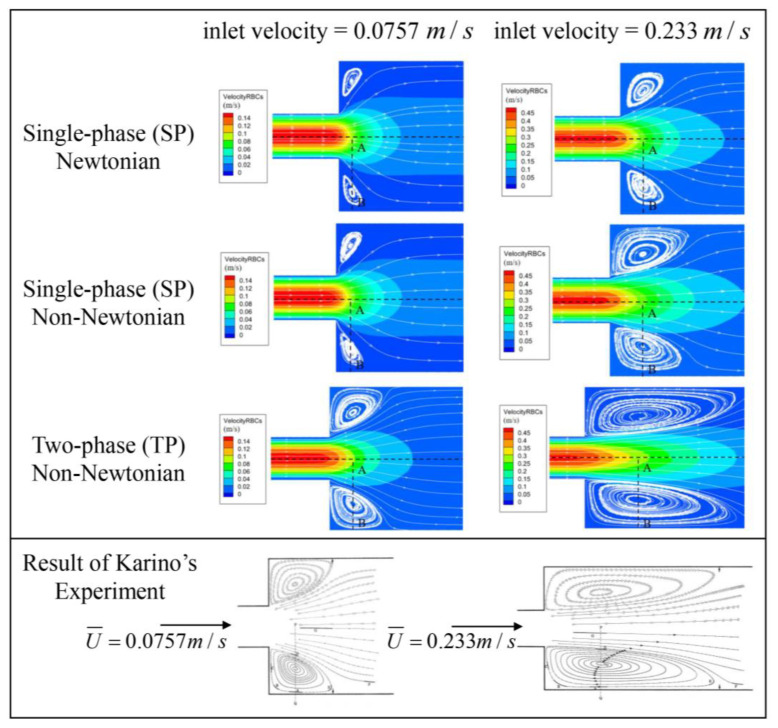
Streamlines and velocity field following sudden expansion [34]. Reprinted with permission from Ref. [34]. 15 July, T. Karino and H. L. Goldsmith.

In the said experiment, diluted blood, containing 1% red blood cells, was directed into the tubular sudden expansion channel at two flow rates. These rates were simulated, corresponding to inlet velocities of 0.0757 m/s and 0.233 m/s. The geometric configuration of the tubular sudden expansion channel is depicted in Figure 5. The same conditions as the experiment are replicated in the simulation to authenticate the blood flow models. Figure 6 showcases the resulting streamlines and velocity fields.

**Figure 7:**
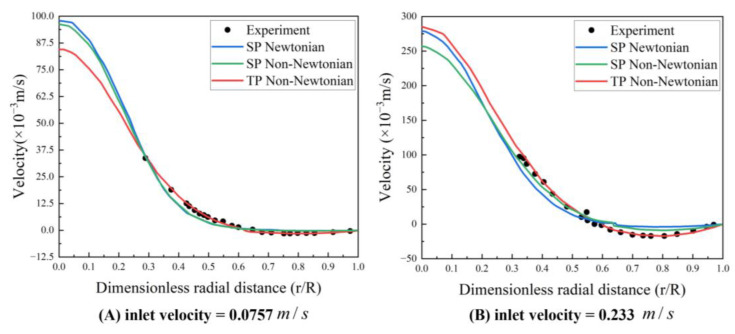
Velocity profile along the A–B line (experimental data from Karino et al. (1977) [34]) Reprinted with permission from Ref. [34]. 15 July 2023, T. Karino and H. L. Goldsmith.

All three models successfully capture the backflow region, albeit with considerable discrepancies. Figure 7 illustrates the velocity distribution along the radial line (A–B) extending from the center of the tube to the wall, crossing the vortex center, and thereby demonstrating a high concurrence between the simulations and experimental data. Even though the three models display conspicuous differences in the streamlines and velocity fields, the velocity trends across all three blood flow models are largely similar. The results from the TP non-Newtonian model align most closely with the experimental value.

### 2.5. Meshing and Grid-Independence Verification

**Figure 8:**
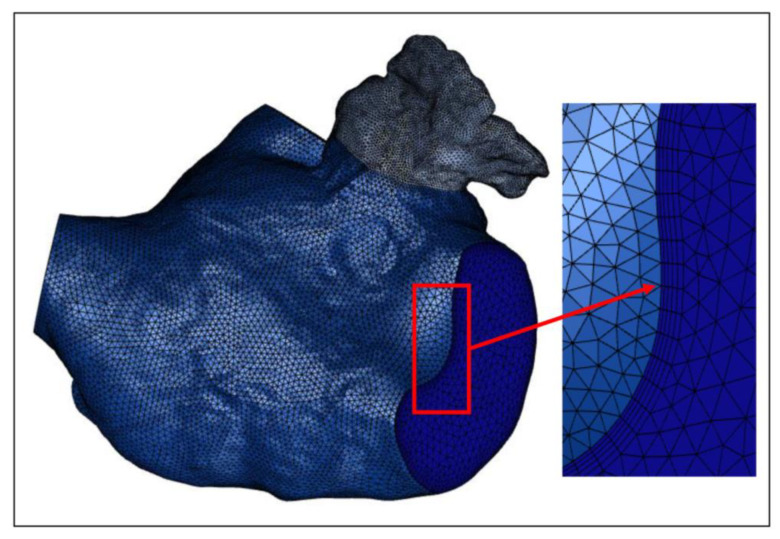
One of the LA meshes.

**Figure 9:**
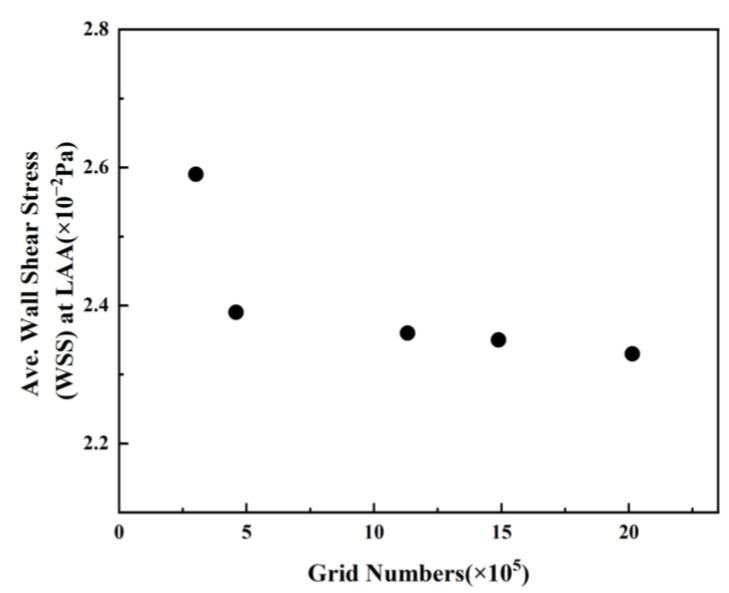
Average *WSS* values at LAA with different grid numbers.

In these simulations, the reconstructed solid model is segmented using a tetrahedral unstructured mesh. The grid at the LAA is refined, and five boundary layers are instituted on the LA wall, as illustrated in Figure 8. For each LA model, five sets of meshes with varying mesh counts are generated, and the average *WSS* at the LAA is employed to verify grid-independence. Figure 9 exhibits the mesh-independent result of one LA model under the TP non-Newtonian blood flow model.

The average *WSS* at the LAA exhibits a downward trend with the augmentation of grid numbers. The declining amplitude of average *WSS* becomes negligible once the grid number surpasses 1,131,541. Consequently, the grid comprising 1,131,541 grid points is selected for the simulation, considering the balance between computational accuracy and efficient allocation of computing resources. Other grid models undergo the same validation process, and the optimal grid is selected for each simulation. The computation is performed on a computer equipped with 48 cores, an Intel(R) Xeon(R) CPU E5-2680 v3 @ 2.50 GHz, and each step takes 12.925 s.

## 3. Result

### 3.1. Analysis of Influence of Blood Flow Models on Flow Field

#### 3.1.1. Effect of Blood Flow Models on Flow Field and Residence Time in Left Atrium

During the early and middle stages of the cardiac cycle, the MV flow rate and velocity remain consistent between SR and AF, and the flow field of SR in the LA closely resembles that of AF. Hence, the data at *t* = 9.9 *T* that were captured in the latter stage of the final cardiac cycle were selected for an analysis and discussion when evaluating the influence of blood flow models on the flow field and *RT* in the LA, where T signifies the duration of a cardiac cycle (0.8 s).

**Figure 10:**
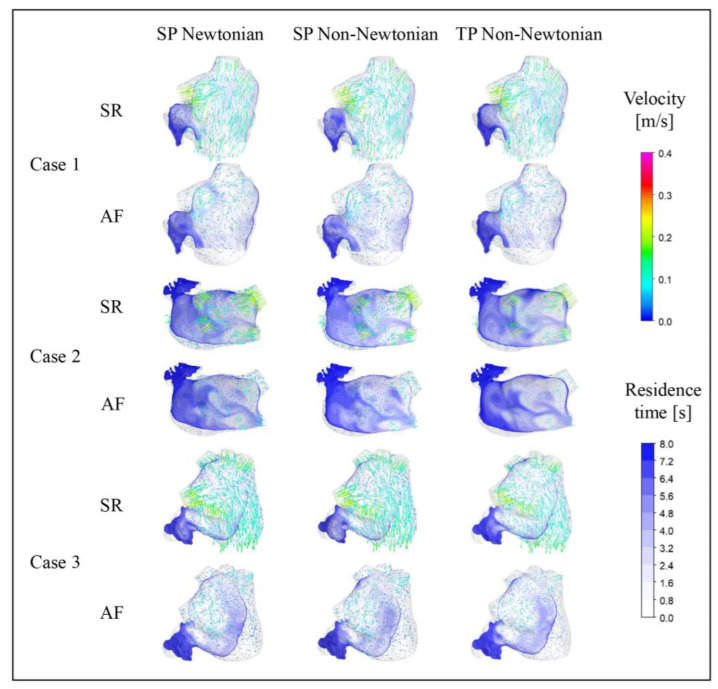
Distribution of blood flow and *RT* in the LA under different blood models at *t* = 9.9 *T*.

**Figure 11:**
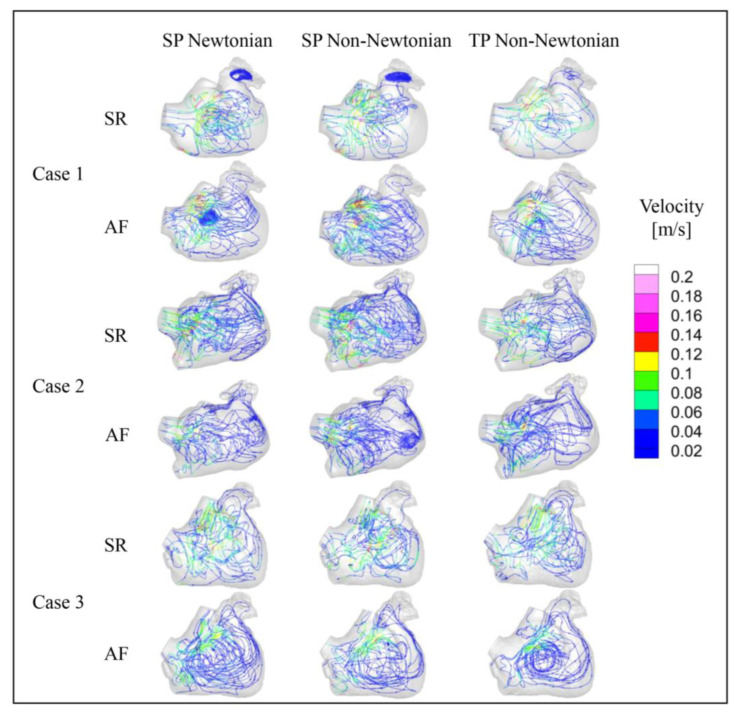
Streamline diagram in the LA under different blood models at *t* = 9.9 *T*.

Figure 10 and Figure 11 illustrate the blood flow velocity, the streamlines in the LA for each case under SR and AF conditions at *t* = 9.9 *T*, and the distribution of blood *RT* in a section of the LA of each case. In these figures, *T* represents the length of a cardiac cycle (0.8 s), and *t* denotes the specific moment of simulation.

The blood flow in the LA, simulated across different blood flow models, is generally consistent across all cases. At *t* = 9.9 *T*, the blood flow velocity in the LA under AF is substantially lower than that under SR. Moreover, the velocity in the LAA is considerably lower than the rest of the LA in both SR and AF conditions. In all scenarios, the areas with high blood flow velocity are situated at the PV inlets and the MV outlet. Interestingly, the internal blood flow velocity in the LA for Case 2 is lower than the other cases under both SR and AF, potentially attributed to a certain degree of atrial enlargement in Case 2.

The distribution of *RT* in a section of the LAA reveals that different blood assumptions exert some influence on blood *RT*. The *RT* distributions simulated by the three blood flow models all exhibit the same pattern, with a high *RT* in the LAA, typically exceeding five cardiac cycles, and the range of high *RT* value is larger in AF. However, numerically, the *RT* in the LAA simulated with the SP non-Newtonian blood flow model is lower, while the TP non-Newtonian blood flow model yields a higher *RT*, particularly in the case of SR.

#### 3.1.2. Effect of Blood Flow Models on Flow Field in Left Atrial Appendage

**Figure 12:**
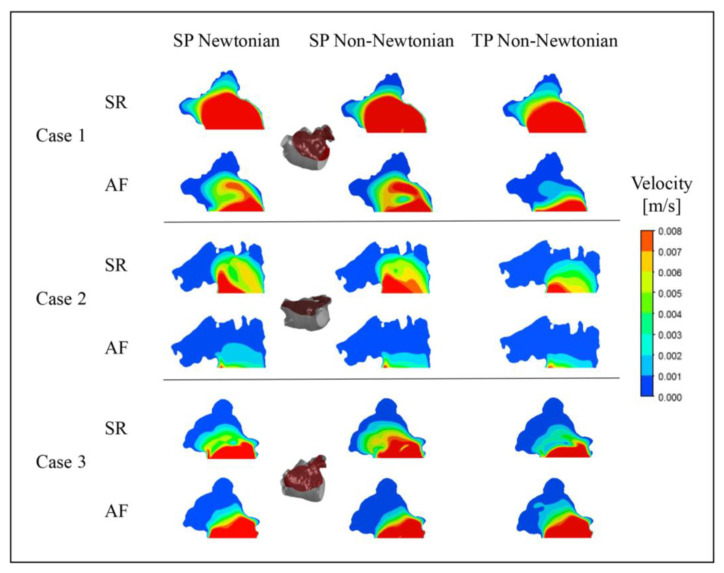
Blood flow velocity in a section of LAA under different blood flow models.

To further examine the blood flow in the LAA under different blood flow models, the blood flow velocity distribution on a section of the LAA at time *t* = 9.9 *T* was selected for analysis, as depicted in Figure 12.

Unexpectedly, in Case 3, the outcome under the TP non-Newtonian blood flow model reveals that the blood velocity in part of the LAA under AF is higher than under SR, while other results align with expectations. This anomaly could be ascribed to variations in the LAA structure, relative position, and blood flow models.

In both SR and AF conditions, the LAA displays a larger range of high velocities under the SP non-Newtonian model, whereas it manifests a smaller range of high velocities under the TP non-Newtonian model. In comparison to AF, the disparity among the results obtained under the three blood flow models is more significant in the SR state, which corroborates the distribution of blood *RT* discussed earlier.

**Figure 13:**
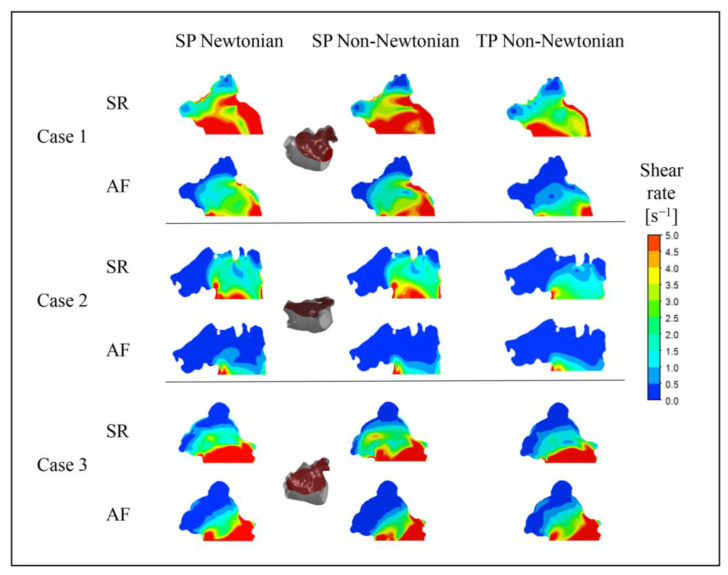
Blood shear rate of a section in LAA under different blood flow models.

In addition, it can be noted that the flow velocity simulated using the SP Newtonian model is higher at the “corner” of the LAA, which is related to the shear rate distributions shown in Figure 13.

### 3.2. The Influence of Blood Flow Models on the Prediction of Thrombosis

#### 3.2.1. Effect of Blood Flow Models on *TAWSS* Value

**Figure 14:**
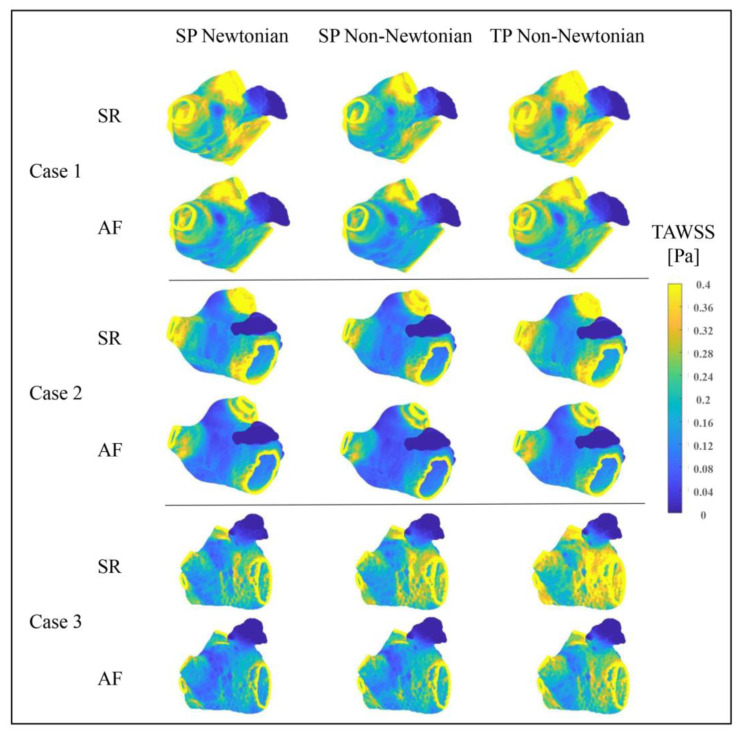
Distributions of *TAWSS* in LA under different blood flow models.

Figure 14 illustrates the distributions of *TAWSS* values in the LA under various blood flow models. The *TAWSS* value depicts the biomechanical effect of *WSS* on the LA wall. Within a particular range, lower *TAWSS* values correspond to reduced flow velocities and an elevated thrombosis risk. The *TAWSS* value distributions in the LA, simulated under diverse blood flow models, are predominantly similar, meaning that, in both SR and AF states, the PVs and MVs possess higher *TAWSS* values, whereas the LAA has a significantly lower *TAWSS* value. Furthermore, when AF occurs, the LA’s primary portion exhibits a substantial decrease in *TAWSS* value, enhancing the thrombosis risk. However, alongside the data in Table 2, the influence of blood flow models on the *TAWSS* value cannot be overlooked.

When comparing the *TAWSS* variation between the SR and AF states under distinct blood flow models, the TP non-Newtonian blood flow model reveals the most significant *TAWSS* value decrease in the LA when AF occurs. This decline better signifies the increased thrombus formation risk in the LA instigated by AF.

Moreover, the average *TAWSS* values in the LA (excluding the LAA) and the LAA, represented in Table 2, suggest that the TP non-Newtonian blood flow model yields higher *TAWSS* values in both SR and AF states. Conversely, the SP non-Newtonian blood flow model results in relatively lower *TAWSS* values. Hence, when evaluating thrombosis risk from the LA wall’s biomechanical effects perspective, the TP non-Newtonian blood flow model typically predicts the minimum thrombosis risk, while the maximum risk is reflected by the SP non-Newtonian blood flow model.

#### 3.2.2. Effect of Blood Flow Models on *OSI* Value

**Figure 15:**
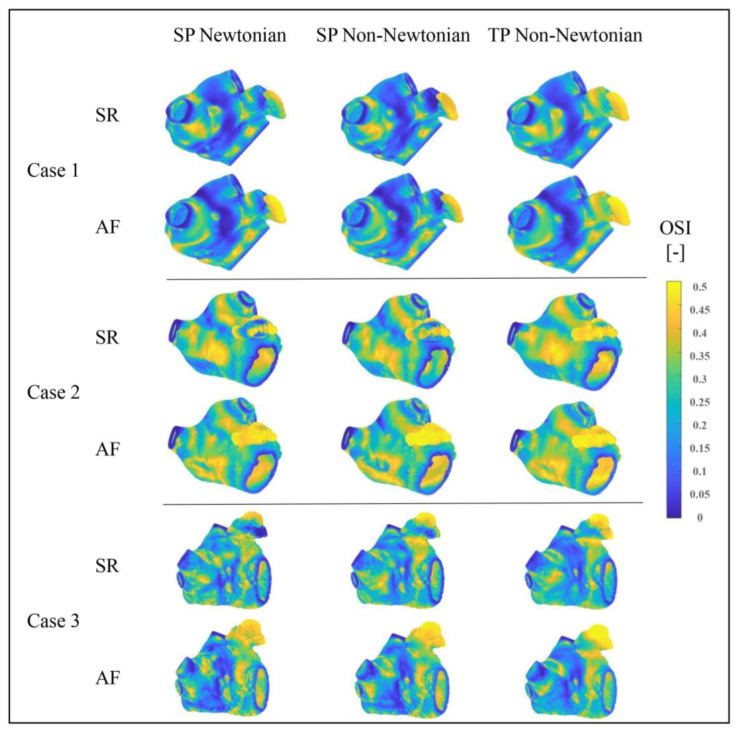
*OSI* distributions of LA under different blood flow models.

Figure 15 exhibits the *OSI* distributions in the LA under varying blood flow models. The *TAWSS* value distributions in the LA, simulated under different blood flow models, are essentially identical, with high *OSI* values mainly located in the LAA, LAA neck, and PV and MV openings. The *OSI* value in the LA’s main part does not significantly change during AF, whereas it does in the LAA.

Numerically, *OSI* is employed to assess thrombosis risk by analyzing the complexity of blood flow pattern changes. Although the overall trend aligns with the results assessed by the *TAWSS* value in the previous section, the thrombosis risk predicted by different blood flow models varies. Among the *OSI* outcomes, the TP non-Newtonian model anticipates the highest thrombosis risk.

Consequently, the *RRT* value and *ECAP* value are scrutinized to further gauge these evaluation indices and blood flow models’ influence on thrombosis prediction.

#### 3.2.3. Effect of Blood Flow Models on *RRT* Value

The *RRT* value merges the *TAWSS* value and *OSI* value, reflecting the blood particles’ *RT* near the wall. A higher *RRT* value accurately signifies a greater thrombosis risk.

**Figure 16:**
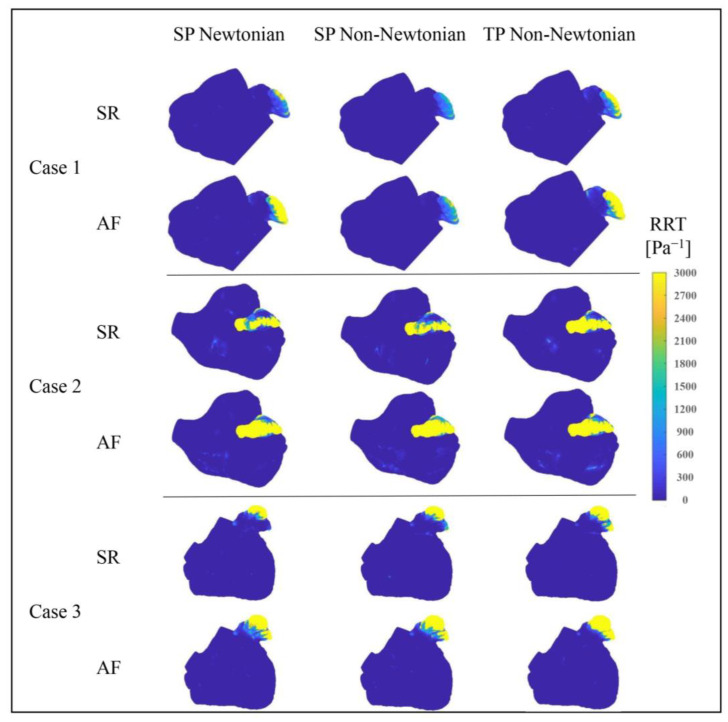
Distributions of *RRT* value in LA under different blood flow models.

Figure 16 depicts the *RRT* value distributions in the LA under varying blood flow models. The *RRT* value distributions, simulated by different blood flow models, are largely similar, with the *RRT* value in the LAA considerably higher than in other regions, suggesting a greater thrombosis risk in the LAA. Moreover, in the state of AF, the LA’s *RRT* value and the high *RRT* value area noticeably increase, contributing to a higher thrombosis risk. Nonetheless, according to the data in Table 3, the influence of the blood flow model on the *RRT* value cannot be dismissed.

Table 3 indicates that the SP non-Newtonian blood flow model yields a slightly higher average *RRT* value for the LA’s main part compared to the other two models, whereas the average *RRT* value of the LAA is much lower. Overall, the predicted thrombosis risk is lower when using the SP non-Newtonian flow model.

Furthermore, it is observed that, except for Case 1 in the SR state and Case 3, the TP non-Newtonian model generates a slightly smaller average *RRT* value in the LA’s main part, while the average *RRT* value in the LAA is comparatively larger. Combined with Figure 16, the TP non-Newtonian model expands the high *RRT* value distribution range at the LAA for Cases 1 and 3 under different heart rhythms, implying a higher thrombosis risk. Therefore, the overall thrombosis risk prediction is greater when utilizing the TP non-Newtonian blood flow model.

#### 3.2.4. Effect of Blood Flow Models on *ECAP* Value

To further substantiate the influence of blood flow models on the predicted risk of thrombosis, the *ECAP* value serves as the evaluative metric in this section. The *ECAP* value amalgamates the *TAWSS* value and *OSI* value and acts as a highly correlated predictor of thrombosis risk: as the *ECAP* value escalates, so does the thrombosis risk.

**Figure 17:**
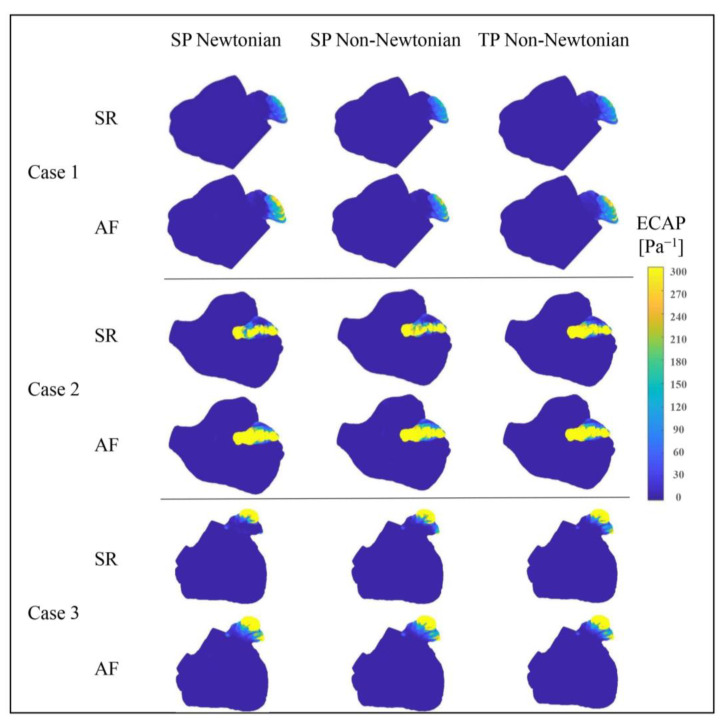
Distributions of *ECAP* value in LA under different blood flow models.

Figure 17 portrays the *ECAP* value distributions in the LA under various blood flow models. The *ECAP* value distributions, simulated by different blood flow models in the LA, appear largely similar, with the LAA’s value notably exceeding that of other LA sections. This implies that the thrombosis risk in the LAA significantly outweighs that in other LA parts.

Additionally, the LA’s *ECAP* value and the high *ECAP* value area prominently increase during AF, resulting in an elevated thrombosis risk. Figure 17 also displays variations among the *ECAP* distributions under different blood flow models. In conjunction with the *ECAP* values in Table 4, it can be deduced that the influence of blood flow models on *ECAP* values cannot be disregarded.

Table 4 reveals that when utilizing the SP non-Newtonian blood flow model, the average *ECAP* value of the LA’s main part (possessing a low thrombosis risk) is marginally higher than that under the other two models. Simultaneously, the LAA’s average *ECAP* value (carrying a high thrombosis risk) is substantially lower.

An anomalous result is observed in Case 1 under the SR state, where the LAA’s average *ECAP* value reaches its minimum under the SP Newton blood flow model. Nevertheless, in other cases, the LAA’s *ECAP* value is relatively smaller when employing the SP non-Newtonian blood flow model. Generally, when the SP non-Newtonian blood flow model is in use, the predicted thrombosis risk is lower.

From the aforementioned analysis, it can be concluded that when different simplified blood models are employed, all thrombosis predictors affirm that the LAA is a high-risk thrombosis area in the LA. Moreover, the thrombosis risk in the LAA amplifies when AF occurs. However, there remain some disparities in the thrombosis risk values calculated by different evaluative indices in the simulation results based on different blood flow models. For instance, under the *RRT* evaluative index, the thrombosis risk predicted by the TP non-Newtonian model is the most pronounced, while the risk predicted by this model is slightly less than the SP Newtonian blood model under the *ECAP* evaluative index.

The *RRT* and *ECAP* values conduct vector integration in the wall shear force analysis. Instead of merely calculating the average value, the unsteady flow nature is considered; thus, they were prevalently employed in current prediction and evaluation. Table 5 showcases the variances in the thrombosis risk evaluation degree of *RRT* and *ECAP* indicators under different blood flow models. Different evaluative indicators respond differently to changes in the blood flow model.

## 4. Discussion

As a common life-threatening disease, AF has brought serious harm to the health of a large number of patients. Nevertheless, the existing clinical stroke risk evaluation criteria primarily depend on empirical factors, thereby frequently resulting in inevitable misinterpretation of stroke risk. Enhancing the precision of stroke risk assessment has emerged as a subject of interest among scholars. This study employs the CFD methodology in conjunction with clinical data to examine the influence of diverse blood flow assumption models on simulation outcomes. The findings of this research furnish a theoretical framework for future endeavors in clinical stroke risk assessment.

The utilization of the fluid–solid interaction approach (FSI) has been widely employed in numerous cardiovascular scenarios. A comparative analysis conducted in a prior investigation, contrasting the computational outcomes of the FSI approach with those of rigid-wall models, revealed a significant impact of arterial wall compliance on the hemodynamic index [35]. Due to the difficulty in obtaining clinical dynamic boundary data and the fact that the LA wall barely contracts under AF, the CFD method was used instead of FSI to study internal blood flow and assess the risk of thrombosis. In contrast to the FSI, the CFD method demonstrates reduced temporal expenditure, albeit occasionally exhibiting diminished precision. Furthermore, the obtained results are in good agreement with the clinical trend, which could be applied to preliminary evaluation and may provide some optimization reference for left atrial appendage occlusion (LAAO). 

However, in order to further reduce the risk of stroke caused by AF in the clinic, the relevant mechanism research is very necessary, such as the mechanism of the thrombosis formation in the LAA. This research can be completed only by deep cooperation between various fields, such as experimental fluid mechanics, CFD, FSI, artificial intelligence (AI), clinical medicine, and so on.

## 5. Limitations

In the current work, we performed analyses on the internal blood flow in the LA of patients with AF, utilizing the CFD methodology. Nevertheless, certain limitations need to be addressed for further investigation:

1. We presumed the LA walls to be rigid. Although this assumption is feasible, particularly in the AF state where the LA wall barely contracts, it differs from the actual scenario. The interaction between the flexible LA wall and blood, along with the heart’s active contraction, can significantly affect the flow pattern. However, due to motion artifacts, dynamic cardiac CT/Magnetic Resonance Imaging (MRI) is not extensively performed on patients [36,37], thus creating a shortage of transient hemodynamic monitoring data and thereby complicating transient CFD simulations. In future research, the interaction between the flexible LA wall and blood will be considered. Currently, we are recruiting volunteers with AF for dynamic cardiac CT data collection.
2. Given the limited conditions, we selected the MV outlet velocity waveform based on international norms to set the MV outlet velocity. Moving forward, the actual MV flow velocity of patients could be acquired as the boundary condition for simulation to procure more precise and individualized numerical simulation results.
3. We simulated only three patients’ cases. It will be crucial to study a larger set of cases in the future to render the conclusions more accurate and reliable.

Stroke can arise from multiple mechanisms wherein the local hemodynamic environment plays a pivotal role in embolism [38]. The transport of cardiogenic plaque (i.e., ‘red’ thrombi) depends on the mechanical properties of the cardio-cerebrovascular system [39]. Thus, future studies could simulate the cardio-cerebrovascular system by using low-dimensional models to obtain more reliable estimates on the risk of embolism and stroke.

## 6. Conclusions

In this study, the LA models of three patients were combined with three different blood flow models to simulate the blood flow in the LA under the SR and AF state. The following conclusions were reached:

1. Blood flow in the LA was roughly the same under both SR and AF when using the three different blood flow models. However, the flow-field details in some parts of LA, such as the “corner” of the LAA, are quite different. Moreover, the *RT* of blood in the LA under the single-phase non-Newtonian blood flow model is the shortest, especially in the SR state, while the *RT* of blood under the two-phase non-Newtonian blood flow is the longest.
2. The *OSI*, *RRT*, and *ECAP* values of the LAA (with high risk of thrombosis) are all relatively lower when using the single-phase non-Newtonian blood flow model, indicating that the risk of thrombosis is lower. On the contrary, when using the two-phase non-Newtonian blood flow model, the *RRT* value in the LAA is relatively higher, causing the predicted risk of thrombosis to be higher.
3. There are some differences in the values of the thrombosis risk calculated by different evaluation indicators in the simulation results obtained by using different blood flow models.

In short, all three blood flow models can simulate the blood flow in the LA in line with the clinical law and predict the risk of thrombosis accordingly, but there are significant differences in the description of the flow-field details and in the judgment of thrombosis risk. In order to obtain more realistic blood flow simulation results, further comparison with clinical data and continuous optimization of the blood flow model are required.

## Figures and Tables

**Table 1:** LA main geometric parameters of 3 patients.

Case	LA Volume (mL)	LAA Volume (mL)	Mitral Orifice Area (cm^2^)	Number of Pulmonary Veins (PVs)
1	136.08	19.33	9.57	4
2	213.40	20.98	9.69	4
3	122.69	19.48	8.70	5

**Table 2:** The average *TAWSS* values of the LA (excluding the LAA) and the LAA.

State	Case	Flow Model	Ave. *TAWSS* in LA (Pa^−1^)	Ave. *TAWSS* in LAA (Pa^−1^)
SR	Case 1	SP Newtonian	0.3588	0.0282
		SP non-Newtonian	*0.2893*	*0.0278*
		TP non-Newtonian	**0.3613**	**0.0290**
	Case 2	SP Newtonian	0.3185	0.0192
		SP non-Newtonian	*0.2566*	*0.0185*
		TP non-Newtonian	**0.3193**	**0.0208**
	Case 3	SP Newtonian	**0.4044**	*0.0331*
		SP non-Newtonian	*0.3262*	0.0337
		TP non-Newtonian	0.4037	**0.0386**
AF	Case 1	SP Newtonian	0.2803	*0.0179*
		SP non-Newtonian	*0.2324*	0.0197
		TP non-Newtonian	**0.2889**	**0.0206**
	Case 2	SP Newtonian	0.2383	0.0120
		SP non-Newtonian	*0.1942*	*0.0112*
		TP non-Newtonian	**0.2421**	**0.0158**
	Case 3	SP Newtonian	0.3089	0.0230
		SP non-Newtonian	*0.2534*	*0.0209*
		TP non-Newtonian	**0.3109**	**0.0289**

Note: The data in bold signify the maximum value obtained under different blood flow models; conversely, the data in italics represent the minimum value.

**Table 3:** The average *RRT* values of the LA (excluding the LAA) and the LAA.

State	Case	Flow Model	Ave. *RRT* Value of LA (Pa^−1^)	Ave. *RRT* Value of LAA (Pa^−1^)
SR	Case 1	SP Newtonian	10.69	*414.00*
		SP non-Newtonian	**12.36**	**1257.73**
		TP non-Newtonian	*9.70*	1167.37
	Case 2	SP Newtonian	18.25	30,534.14
		SP non-Newtonian	**21.82**	*17,758.14*
		TP non-Newtonian	*17.32*	**35,697.30**
	Case 3	SP Newtonian	11.01	**41,553.43**
		SP non-Newtonian	**12.59**	*4829.39*
		TP non-Newtonian	*9.48*	11,569.01
AF	Case 1	SP Newtonian	16.34	4212.30
		SP non-Newtonian	**16.68**	*846.95*
		TP non-Newtonian	*13.48*	**4272.21**
		SP Newtonian	*27.54*	*118,042.04*
	Case 2	SP non-Newtonian	**34.37**	131,275.75
		TP non-Newtonian	30.95	**153,265.30**
	Case 3	SP Newtonian	13.19	**31,588.81**
		SP non-Newtonian	**16.08**	*6819.30*
		TP non-Newtonian	*12.26*	21,834.54

Note: The data in bold signify the maximum value obtained under different blood flow models; conversely, the data in italics represent the minimum value.

**Table 4:** Average *ECAP* values of the LA (excluding the LAA) and the LAA.

State	Case	Flow Model	Ave. *ECAP* Value of LA (Pa^−1^)	Ave. *ECAP* Value of LAA (Pa^−1^)
SR	Case 1	SP Newtonian	0.93	*37.24*
		SP non-Newtonian	**1.12**	41.05
		TP non-Newtonian	*0.89*	**42.72**
	Case 2	SP Newtonian	1.51	706.80
		SP non-Newtonian	**1.80**	*480.37*
		TP non-Newtonian	*1.38*	665.78
	Case 3	SP Newtonian	0.96	**138.88**
		SP non-Newtonian	**1.09**	*81.21*
		TP non-Newtonian	*0.86*	105.32
AF	Case 1	SP Newtonian	1.34	**77.77**
		SP non-Newtonian	**1.51**	*49.78*
		TP non-Newtonian	*1.19*	69.22
	Case 2	SP Newtonian	2.22	**1119.29**
		SP non-Newtonian	**2.59**	*794.72*
		TP non-Newtonian	*2.04*	938.10
	Case 3	SP Newtonian	1.14	**197.79**
		SP non-Newtonian	**1.35**	*137.74*
		TP non-Newtonian	*1.02*	157.88

Note: The data in bold signify the maximum value obtained under different blood flow models; conversely, the data in italics represent the minimum value.

**Table 5:** Evaluation of thrombosis risk by *RRT* and *ECAP* under different blood flow models.

Indicators	Flow Model	Predicted Thrombosis Risk
*RRT*	SP Newtonian	Medium
	SP non-Newtonian	Lower
	TP non-Newtonian	Higher
*ECAP*	SP Newtonian	Higher
	SP non-Newtonian	Lower
	TP non-Newtonian	Medium

## Data Availability

Not applicable.
